# Difficult ventilation with suspected laryngospasm in patients undergoing pulsed field ablation for atrial fibrillation under general anesthesia using laryngeal mask airway: A case series

**DOI:** 10.1016/j.hrcr.2025.08.021

**Published:** 2025-11-15

**Authors:** Kazumasa Suga, Hiroyuki Kato, Hisashi Murakami, Satoshi Yanagisawa, Yasuya Inden, Toyoaki Murohara

**Affiliations:** 1 Department of Cardiology, Japan Community Healthcare Organization Chukyo Hospital, Nagoya, Japan; 2 Department of Cardiology, Nagoya University Graduate School of Medicine, Nagoya, Japan

**Keywords:** Atrial fibrillation, Difficult ventilation, General anesthesia, Laryngeal mask airway, Laryngospasm, Pulsed field ablation

**Key Teaching Points**

• Pulmonary vein isolation using pulsed field ablation (PFA) for atrial fibrillation under general anesthesia with a laryngeal mask airway and no neuromuscular blocking agents (NMBAs) can cause sudden ventilation difficulty owing to suspected laryngospasm, even under adequate anesthetic depth.
• PFA may induce difficult ventilation suspected to result from laryngospasm via vagal reflexes, even when applied at sites distant from the recurrent laryngeal nerve.
• NMBAs are effective in both the prevention and treatment of difficult ventilation suspected to result from laryngospasm during PFA.

## Introduction

Pulmonary vein isolation (PVI) is an established treatment for atrial fibrillation (AF).^1^ Recently, pulsed field ablation (PFA) has emerged as a novel, nonthermal technique delivering high-voltage, ultrashort-duration electrical pulses that induce irreversible electroporation in myocardial cells, enabling selective tissue ablation. Compared with conventional thermal sources such as radiofrequency or cryoablation, PFA may reduce injury to adjacent structures such as the esophagus and phrenic nerve.^2^ Despite promising safety features, PFA is relatively new, and its complications and long-term safety remain under investigation.

High-voltage pulses during PFA can cause significant muscular and diaphragmatic contractions, often necessitating deep sedation or general anesthesia.^3^ For airway management during catheter ablation for AF under general anesthesia, a laryngeal mask airway (LMA) is widely used owing to its lower invasiveness and ease of insertion compared with endotracheal intubation.^4^ However, using an LMA without neuromuscular blocking agents (NMBAs) during general anesthesia poses a risk of sudden difficult ventilation, primarily owing to laryngospasm.^5^

We report 3 cases of suspected laryngospasm during PVI with PFA, all performed under general anesthesia using an LMA.

## Case reports

This section describes 3 cases of difficult ventilation with suspected laryngospasm during PVI using PFA. None of the patients had a history of respiratory disease. The *anesthetic protocol* was standardized as follows: induction with propofol (2.0–2.5 mg/kg) and fentanyl (100 μg bolus), followed by an initial loading dose of dexmedetomidine (6 μg/(kg·h) over 10 minutes). Maintenance anesthesia consisted of propofol (4.0–10.0 mg/(kg·h)), fentanyl (50 μg bolus every 30 minutes), and dexmedetomidine (0.4 μg/(kg·h)). Anesthetic depth was continuously monitored using a bispectral index (BIS) monitor (Covidien). Anesthetic doses were adjusted as needed on the basis of the patient’s individual clinical response and BIS monitoring. Although general anesthesia was induced using an LMA without prophylactic NMBAs in cases 1 and 2, prophylactic NMBA was administered in case 3. Atropine sulfate (0.5 mg) was administered as a bolus before PFA initiation. The PulseSelect PFA system (Medtronic) was used for PVI, and a multipolar catheter was introduced into the coronary sinus via the left subclavian vein. Transseptal puncture was performed under intracardiac echocardiographic guidance. The FlexCath Contour sheath (Medtronic) was then advanced into the left atrium (LA) over a guidewire. The PVI protocol using the PulseSelect system was consistent across all cases, utilizing the manufacturer’s predefined pulse settings. A set of 4 pulses was delivered at the distal portion of the pulmonary vein (PV) ostium, followed by 4 additional pulses at an antral position. After each pulse delivery, the catheter was rotated 90° to achieve full circumferential PVI. The sequence of PVI for each PV was at the operator’s discretion, and additional applications were performed as needed. For the LA posterior wall, linear and continuous applications were delivered along the LA roof and floor lines. Patient characteristics and procedural details for all 3 cases are summarized in Tables 1 and 2. Written informed consent was obtained from all patients for the AF ablation procedure as well as for publication of this case series, including the use of anonymized clinical data and any associated images or videos.

**Table 1:** Patient and clinical characteristics of the 3 cases

Case no.	Age (y)/sex	Height (cm)	Weight (kg)	BMI (kg/m^2^)	AF type	LVEF (%)	LAD (mm)	LAVI (mL/m^2^)	CHADS_2_ score	Anticoagulant (type, dose)	Creatinine (mg/dL)	BNP (pg/mL)
1	67/male	166.5	87.6	31.6	Persistent	68	56	64	3 (CHF, HT, DM)	Rivaroxaban (15 mg) once daily	0.7	166.7
2	68/female	160.9	52.8	20.4	Paroxysmal	60	37	53	1 (HT)	Rivaroxaban (15 mg) once daily	0.5	169.2
3	52/male	176.5	119.4	38.4	Long-standing persistent	62	46	21	1 (HT)	Apixaban (5 mg) twice daily	1.2	17.7

AF = atrial fibrillation; BMI = body mass index; BNP = brain natriuretic peptide; CHF = congestive heart failure; DM = diabetes mellitus; HT = hypertension; LAD = left atrial diameter; LAVI = left atrial volume index; LVEF = left ventricular ejection fraction.

**Table 2:** Anesthetic dose, difficult ventilation events, and management details of the 3 cases

Event site	NMBA preloaded	Minimum SpO_2_	BIS before PFA	Intervention	Outcome	Total anesthetic dose
LSPV LIPV RSPV	None	84%	45	LMA adjustment, manual ventilation, propofol bolus (50 mg)	Resolved within 3 min; mild events (<30 s) still recurred even after increasing the propofol dose (BIS <30)	Propofol (1012 mg), fentanyl (200 μg), dex (102 μg)
LSPV	None	81%	48	Rocuronium bolus (50 mg)	Resolved after NMBA; no recurrence	Propofol (740 mg), fentanyl (300 μg), dex (73 μg), and rocuronium (50 mg)
LIPV	Rocuronium (70 mg) (presumed recovered [TOF count 4/4] at the event)	78%	37	Rocuronium bolus (50 mg)	Resolved after NMBA; no recurrence	Propofol (1386 mg), fentanyl (250 μg), dex (158 μg), and rocuronium (120 mg)

BIS = bispectral index; Dex = dexmedetomidine; LIPV = left inferior pulmonary vein; LMA = laryngeal mask airway; LSPV = left superior pulmonary vein; NMBA = neuromuscular blocking agent; PFA = pulsed field ablation; RSPV = right superior pulmonary vein; SpO_2_ = peripheral oxygen saturation; TOF = train-of-four.

### Case 1

A 67-year-old man was admitted for catheter ablation of persistent AF lasting 4 months. After induction of general anesthesia, a size 5 i-gel LMA (Intersurgical Ltd.) was successfully inserted on the first attempt. Adequate ventilation was maintained until PFA initiation. Immediately after the first PFA application to the left superior PV (LSPV), the patient experienced a forceful cough followed by complete loss of the capnographic waveform. The BIS value was 45, and the ventilator alarm indicated hypoventilation. Initial interventions, including LMA repositioning, bag-valve-mask ventilation, and airway suctioning for potential secretions, were ineffective. Although the cough reflex subsided rapidly and no wheezing was detected on auscultation, mechanical ventilation remained unachievable owing to marked airway resistance. Peripheral oxygen saturation (SpO_2_) dropped to 84%. A 50 mg bolus of propofol was administered, leading to gradual recovery of ventilation within 3 minutes. Despite deepening anesthesia (propofol [500 mg/h]; targeting BIS <30), hypoventilation due to increased airway resistance was repeatedly observed (4 times during the LSPV, 2 times during the left inferior PV [LIPV], and once during the right superior PV). Although SpO_2_ consistently remained at or above 90% during these subsequent episodes, minor oxygen desaturation still occurred despite the increased anesthetic depth, with each episode resolving spontaneously within 30 seconds. PVI was successfully completed with a total of 36 PFA applications over 135 minutes. Emergence from anesthesia was uneventful, and no postoperative neurological or respiratory complications were noted.

### Case 2

A 68-year-old woman with a history of radiofrequency ablation for paroxysmal AF 4 years prior presented with recurrence and underwent PFA. A size 4 i-gel LMA was inserted uneventfully. Adequate ventilation was maintained until PFA initiation. Reconnection of the LSPV and LIPV was observed. Immediately after PFA application to the LSPV, the patient experienced violent coughing with body movements, followed by complete ventilatory failure and disappearance of the capnogram. The BIS value was 48, and SpO_2_ decreased to 81%. Breath sounds were absent on auscultation. LMA repositioning and bag-valve-mask ventilation were unsuccessful. Intravenous administration of rocuronium (50 mg) gradually restored adequate ventilation within 3 minutes. Muscle relaxation was confirmed by train-of-four (TOF) monitoring (ToFscan, IDMed) at the left adductor pollicis (ulnar nerve stimulation; TOF count 0/4). No further episodes of difficult ventilation occurred. Reisolation of the left PVs and LA posterior wall using PFA were successfully achieved. Overall, 25 PFA applications were performed over a 145-minute procedure. After the procedure, sugammadex (200 mg) was administered to antagonize the effect of rocuronium. Emergence from anesthesia and extubation were uneventful. No postoperative neurological or respiratory complications were observed.

### Case 3

A 52-year-old man with long-standing persistent AF for over 3 years underwent PFA. A size 5 i-gel LMA was inserted without difficulty, and ventilation was adequate before PFA initiation. Electroanatomic mapping was performed using an HD Grid catheter (Abbott). Rocuronium (70 mg) was administered 5 minutes before the first PFA application, resulting in complete muscle relaxation (TOF count 0/4). However, approximately 40 minutes after NMBA administration, with presumed neuromuscular recovery (TOF count 4/4), complete ventilatory failure occurred immediately after PFA application to the LIPV antrum. The BIS value was 37, and SpO_2_ decreased to 78%. Breathing sounds were absent. LMA repositioning and manual ventilation were ineffective. An additional 50 mg of rocuronium was administered, achieving a TOF count of 0/4, which restored ventilation within 2 minutes, with full recovery by 3 minutes (Supplemental Video 1). No further episodes of difficult ventilation occurred, including during additional PFA at the same site. A total of 37 PFA applications were performed over 148 minutes. After the procedure, the reversal of rocuronium was performed with sugammadex (400 mg). Emergence from anesthesia and extubation were uneventful, and no postoperative neurological or respiratory complications were observed.

## Discussion

We report 3 consecutive cases of unexpected and severe difficult ventilation during PVI using PFA under general anesthesia with an LMA. Difficult ventilation was observed in cases where NMBAs had not been administered or their effects had worn off. Although PFA is rapidly gaining popularity as a nonthermal ablation modality for AF, anesthesia-related complications remain underreported. This case series highlights a potentially significant yet underrecognized airway complication during PFA and offers insights into its effective management.

Differential diagnoses for sudden difficult ventilation with an LMA include malposition, airway obstruction (eg, tongue base collapse, epiglottic downfolding, secretions, or blood), bronchospasm, pulmonary pathology (eg, pneumothorax or pulmonary edema), or ventilator malfunction.^6^ In our cases, the LMA functioned properly before the events, and repositioning or suctioning failed to restore ventilation. The absence of wheezing and complete loss of the capnographic waveform indicated near-total airway obstruction. β-Agonists were not administered during these episodes. Importantly, ventilation promptly resumed after NMBA administration. NMBAs effectively relieve skeletal muscle spasm but do not directly alleviate the smooth muscle spasm of bronchospasm, for which β-agonists are the primary treatment.^7^ Therefore, this clinical response strongly supports laryngospasm as the underlying cause—an involuntary, reflexive, and sustained vocal cord closure mediated by intrinsic laryngeal muscles and the recurrent laryngeal nerve.^8^ Laryngospasm can rapidly cause hypoxemia, bradycardia, and, in severe cases, hypoxic brain injury, cardiac arrest, or death.

LMA use under insufficient anesthetic depth or without NMBAs is recognized as a risk factor for laryngospasm.^5^^,^^6^ Propofol is effective for treating laryngospasm,^6^ and dexmedetomidine reportedly reduces airway reflex stimulation.^9^ Atropine is often used as a premedication for general anesthesia to prevent vagal reflexes. However, despite increasing the propofol infusion and attempting to lower the BIS value, difficult ventilation could not be prevented in our cases.

Obesity, airway hyperreactivity, and gastroesophageal reflux are established risk factors for LMA-related airway difficulty and laryngospasm.^6^ Notably, cases 1 and 3 in our series were categorized as obese (Table 1). Therefore, patients with these underlying risk factors may be at an increased risk of laryngospasm after PFA, and endotracheal intubation may be a safer airway management strategy in such cases.

**Figure 1:**
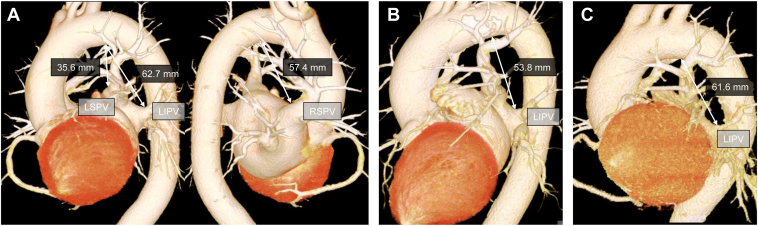
Anatomical distances from the PV ostia to the inferior border of the aortic arch, assessed by 3-dimensional CT. **A:** Case 1 image shows distances from the LSPV (35.6 mm), LIPV (62.7 mm), and RSPV (57.4 mm) ostia to the inferior border of the aortic arch. This border approximates the left recurrent laryngeal nerve path. **B:** Case 2 image shows the LIPV–aortic arch distance as 53.8 mm. **C:** Case 3 image shows the LIPV–aortic arch distance as 61.6 mm. CT = computed tomography; LIPV = left inferior pulmonary vein; LSPV = left superior pulmonary vein; PV = pulmonary vein; RSPV = right superior pulmonary vein.

Although PFA is considered highly selective for cardiomyocytes, effects such as diaphragmatic contraction due to phrenic nerve stimulation and coughing from bronchial irritation have been reported.^10^ However, its effects on upper airway reflexes during general anesthesia remain poorly characterized. Żuchowski et al^11^ reported a case of laryngospasm during PFA of the LSPV under general anesthesia with an LMA. They hypothesized that the mechanism involved direct stimulation of the recurrent laryngeal or vagus nerve. Computed tomography revealed a distance of 34.2 mm between the LSPV and the inferior margin of the aortic arch, which is presumed to approximate the course of the left recurrent laryngeal nerve. In contrast, in our cases, difficult ventilation with suspected laryngospasm occurred not only during LSPV ablation but also at other PV locations. Furthermore, the distances from PVs—except the LSPV—to the inferior margin of the aortic arch ranged from 53.8 to 62.7 mm (Figure 1). These distances were considerably longer than those reported in the previous study. Therefore, given the anatomical distances between these PVs and the left recurrent laryngeal nerve, direct stimulation of the nerve trunk is unlikely. We speculate that an alternative mechanism may involve indirect activation of afferent vagal fibers around the heart, bronchi, and esophagus, subsequently triggering reflexive laryngeal muscle contraction via central neural circuits. This hypothesis aligns with reports indicating that laryngospasm can be induced by distant visceral stimulation via central reflex pathways.^6^ Furthermore, experimental evidence in dogs has demonstrated that stimulation of distal esophageal afferents can trigger laryngospasm, which is abolished by bilateral vagotomy.^12^ Alternatively, PFA-induced muscle contractions and coughing may cause significant patient movement, potentially leading to LMA displacement and subsequent upper airway irritation, which, in turn, could trigger laryngospasm. Further research is required to fully elucidate the precise mechanisms of difficult ventilation, including laryngospasm, induced by PFA.

We used the PulseSelect PFA system in all cases of our series. This device is categorized as a wide-area PFA system and may generate a more extensive high-voltage electrical field. Recently, focal PFA systems have been introduced into clinical practice.^13^ Their more limited electrical fields may reduce the risk of unintended stimulation of vagal afferent fibers or adjacent structures involved in airway reflexes. Further studies are warranted to directly compare the risk of collateral effects between wide-area and focal PFA systems.

The key clinical implication of this case series is that difficult ventilation with suspected laryngospasm can occur during PFA under general anesthesia with an LMA, even with adequate anesthetic depth. Therefore, we strongly recommend concomitant NMBA administration during general anesthesia with an LMA for PFA procedures, as prompt NMBA administration remains the most reliable treatment for relieving laryngospasm.^8^ Continuous capnographic monitoring is essential for early detection of ventilatory failure, and advanced airway equipment, including endotracheal intubation, must be immediately available.

This case series has some limitations. Our observations are limited to cases involving LMA use, and we did not encounter difficult ventilation with face mask ventilation. Laryngospasm was not directly confirmed via fiberoptic visualization. Additionally, all cases involved the PulseSelect PFA system, and it remains unknown whether other PFA systems carry similar risks.

## Conclusion

During PFA-guided PVI under general anesthesia with an LMA, difficult ventilation can occur even when adequate anesthetic depth is maintained. The most likely cause is laryngospasm-induced airway obstruction. Therefore, the strategic use of prophylactic or readily available NMBA administration is strongly recommended to ensure airway safety during PFA.

## Disclosures

The authors have no conflicts of interest to disclose.
