# Supplementary material for: Difficult ventilation with suspected laryngospasm in patients undergoing pulsed field ablation for atrial fibrillation under general anesthesia using laryngeal mask airway: A case series
Source: HeartRhythm Case Rep. 2025 Nov 15;11(12):1271–5. doi: 10.1016/j.hrcr.2025.08.021 (PMC12805282; doi:10.1016/j.hrcr.2025.08.021)
Supplement: Supplementary Material [file mmc1.docx]

**Supplemental video. Fluoroscopic assessment of diaphragmatic motion and ventilation during PFA in Case 3**

Sequential fluoroscopic clips demonstrate changes in diaphragmatic motion and ventilation during LIPV PFA:

1. Before PFA: Normal diaphragmatic excursion is observed during mechanical ventilation.
2. During initial PFA: Coughing is induced, resulting in sustained diaphragmatic elevation.
3. Post-PFA delivery: Diaphragmatic motion is minimal due to impaired mechanical ventilation.
4. Two minutes post-PFA: Manual ventilation gradually restores airflow, and diaphragmatic motion reappears.
5. Three minutes post-PFA: Ventilation and diaphragmatic motion return to baseline.

LIPV, left inferior pulmonary vein; PFA, pulsed field ablation.
